# Express Method for Assessing Performance of Lubricant Compositions Containing Nano-Additives Used for Wheel–Rail Pairs

**DOI:** 10.3390/ma17112499

**Published:** 2024-05-22

**Authors:** Valerii Kosarchuk, Mykola Chausov, Volodymyr Tverdomed, Kostyantyn Lopatko, Vaidas Lukoševičius

**Affiliations:** 1 Faculty of Infrastructure and Rolling Stock of Railways, State University of Infrastructure and Technologies, Kyrylivska Str. 9, 04071 Kyiv, Ukraine; kosarchuk_vv@gsuite.duit.edu.ua; 2 Faculty of Mechanical Engineering and Design, National University of Life and Environmental Sciences of Ukraine, Heroiv Oborony Str. 15, 03041 Kyiv, Ukraine; m.g.chausov@gmail.com (M.C.);; 3 Faculty of Mechanical Engineering and Design, Kaunas University of Technology, Studentu Str. 56, 51424 Kaunas, Lithuania

**Keywords:** sliding friction, hardness, lubricant, nano-additives, wear resistance, wheel–rail pair

## Abstract

An express method for assessing the effectiveness of lubricating compositions with nano-additives of various chemical compositions is proposed, and a joint analysis of experimental data on the changes in the value of wear and the level of damage to the surface layers of metallic friction pairs was performed. The variation in the current relative hardness of the sample’s surface, the variation in the current relative material damage level, the current value of wear, and the current level of the coefficient of friction were chosen as the key parameters to conduct a performance assessment. The level of material damage in the contact zone was determined using the parameters of the statistical law of hardness value scattering. Based on an analysis of data in the literature, it was observed that the structural changes occurring in metallic materials during long-term, cyclic, static, and frictional loading are correlated with changes in the statistical characteristics of the hardness scattering results. An experimental substantiation of the proposed method was carried out for steel-sliding friction pairs using lubricating compositions based on Greaseline Lithium BIO Rail 000 oil manufactured by AIMOL with nano-additives of copper, magnesium and aluminum alloys, graphite, and two grades of medium-carbon steel. According to the system of indicators presented in this research, the greatest efficiency (in terms of increasing the wear resistance of friction steel pairs) was achieved with lubricating compositions including nano-powder additives made of steel, which have lower hardness. For the friction experiments, where the determining factor was abrasive wear, such lubricants ensured minimal damage and wear to the friction surface, while the value of the friction coefficient was maintained at a level that is optimal for wheel–rail friction pairs.

## 1. Introduction

One of the main problems faced by transport structures is the wear of friction unit parts. Promising directions in solving this problem include methods for modifying the contact surfaces of parts using various types of coatings or lubricants [1,2]. The use of coatings is most effective for friction pairs of the “shaft-bushing” type, as well as joints with an interference fit. In this case, the correct choice of material and coating technology can prevent the accumulation of defects and the destruction of such friction pairs [3,4]. For open friction pairs of the “wheel–rail” type, the use of lubricants is more economically effective. Wear is one of the main causes of the accumulation of defects in the wheels of rails and rolling stock. Open friction pairs of the “wheel–rail” type are known [5,6,7] to be highly susceptible to abrasive and fatigue wear. Fatigue damage and abrasive wear accumulate simultaneously, and are associated with the intensive plastic strain of the upper layers of friction pair materials as a result of a high level of contact stresses. While the abrasive wear of rails is mainly related to the failure of materials due to wheel sliding and the presence of solid particles in the contact zone, fatigue wear depends on the cyclic nature of the load and the intensity of damage accumulation in the microstructure of the rail materials. The process of rail wear depends on many factors (e.g., the relative hardness of the wheels and rails, the velocity of movement, the geometric shape of the rolling surfaces of rails and wheels, the track geometry, the quality of materials, the technical condition of the track and rolling stock, and so on), some of which are fairly difficult to account for formally under operating conditions. Consequently, the possibilities of solving this problem theoretically—for example, through mathematical modeling—are limited.

Lubricating the working surfaces of rails and wheels is considered to be the main means of abrasive wear control [7]. The introduction of lubricating materials into the friction zone leads to a considerable change in the nature and intensity of friction processes, wear, and defect formation. Moreover, these processes can be controlled to some extent through the use of lubricants and additives having different chemical compositions. Many researchers have explored the options for improving the performance of industrial lubricating oils using additives, in particular, with nano-materials of different chemical compositions [8,9,10,11,12]. Natural or synthetic metal-containing compounds (serpentine, aluminosilicates, dolomite, magnetite, fatty acid polyvalent-metal salts, and so on), nano-powders of various metals and alloys, and carbon-based materials (graphene, graphite, and diamond) are commonly used as nano-additives.

Industrial oils, in the form of thickened petroleum or mineral oil compositions containing special multifunctional additives, are often used to lubricate rails and wheels. The lubricants used for this purpose have a viscosity of 240 to 350 MPa in the temperature range from 20 to 60 °C and have a high level of adhesion. The tribological characteristics of friction pairs are affected by even an insignificant content of nano-material additives (0.1 to 10 wt.%), while the basic physical properties (density, adhesive characteristics, environmental resistance, application temperature range, and so on) of these types of lubricants remain almost unchanged.

The properties of nanoparticles and their surface activity are influenced by their size and morphology, which may differ depending on the production methods. At present, quite a few technologies have been developed for the industrial production of nano-additives, including the following common physical methods: gas-phase synthesis, the exploding wire method, cathode sputtering, mechanical and ultrasonic dispersion, and a variety of chemical and combined methods [13,14,15].

The use of multiple methods for producing lubricating compositions has led to issues related to choosing the best composition for specific applications, as no universal criteria for assessing the performance of lubricating compositions have been established to date. It should be noted that the performance of lubricating oils and additives depends not only on their chemical composition, but also on the type of friction pair, the service conditions of tribounits, the compatibility between the components of the lubricating composition and the friction pair materials, and so on. The criteria for assessing the performance of lubricating compositions should be based on an estimation of the working characteristics of a particular friction pair. For many tribounit types, the wear resistance can be improved significantly only when the friction coefficient is reduced significantly as well. Therefore, in practice, a simplified method for assessing the effectiveness of different types of lubricating oils is often beneficial. In particular, a high-performance oil is a lubricating oil that provides a minimum friction coefficient and maximum stability of the lubricating film under specific temperature–force operating conditions of the friction pair. However, the value of the friction coefficient changes throughout the experiment as a result of micro-relief deformations as well as changes in the temperature, damage accumulation, and physical and mechanical properties of the lubricant and the materials of the constituent elements of the friction pair. Therefore, not only laboratory test results, but also in situ data should be used to evaluate the performance with respect to a lubricant’s composition. Different methods for assessing the performance of lubricating oils, which often combine laboratory and in situ experiments [16,17,18], have been specifically developed for respective industries or types of transport equipment. However, in the case of wheel–rail friction pairs, the possibility of conducting in situ experiments is extremely limited due to the uncertainty of the load conditions, the influence of the environment, and the long duration and cost of work. In [19], it was suggested that laboratory investigations should be carried out under a comprehensive procedure that includes wear experiments applying slipping friction. The authors consider this experimental methodology to be the most appropriate, as it facilitates high reproducibility of the results, a short duration, and a moderate research cost. It should be noted that certain characteristics of in situ conditions are difficult to reproduce in these kinds of experiments (for example, the dependence of the effective friction coefficient on the change in the shape of the working surfaces of rails and wheels due to wear) [20].

In most cases, the performance of lubricating compositions is assessed using several single (value of wear, value of friction coefficient) or integrated indicators [21,22]. Thus, the authors of [22] proposed a special algorithm to evaluate the effectiveness of lubricants. This algorithm is based on verifying the compliance of the specific thickness criteria of the lubricant layer or the boundary temperature of the lubricant performance. In this case, both the temperature dependence of the lubricating material’s kinematic viscosity and certain characteristics of the friction pair (the provided radius of curvature of the contact pair, the provided modulus of elasticity of the materials, the average roughness parameter of the friction surfaces, the temperature, and the value of the applied load) are considered. However, the effect of lubrication on the degree of damage to contact surfaces and the wear rate are not taken into account. When several lubricants of the same type (e.g., lubricating compositions based on industrial grease containing different nano-additives) are assessed, the values of the criteria mentioned above may be the same or close, and the reliability of this methodology would be questionable.

Another method of assessing lubrication performance that accounts for changes in the elastic–plastic properties of the surface layers of parts due to friction was proposed in [23]. In this method, the lubricant’s performance is determined by the change, ∆*g*, in the specific energy of the plastic deformation of the dry and lubricated surface, which is considered to be the activation energy of the thermomechanical failure of the lubricant. Positive ∆*g* values indicate the strengthening of the friction surface, while negative values signal the plasticizing of the surface, and zero values indicate the absence of an impact. The value of the specific energy of the plastic strain is determined based on the results of a sclerometer or surface micro-indentation test. However, as demonstrated in the following sections, these integral characteristics of mechanical properties are largely insufficiently sensitive to changing structural conditions.

One of the characteristics of wheel–rail friction pairs is the need to maintain a certain coefficient of friction (0.2<f<0.35). Its reduction to less than 0.2 creates a movement safety risk, as traction modes may become non-stationary, which may lead to derailment on curved sections of the track [7]. Another argument in favor of maintaining the friction coefficient within these limits is the idea that moderate wear to the working surfaces of the rails and wheels acts as a preventive measure against the development of surface fatigue cracks. A difference in the hardness of the contacting surfaces is also an important characteristic in these kinds of tribosystems. The hardness may differ for different types of railway track, and it can be determined using the respective national regulations (the ratio of the hardness of new wheels to the hardness of new rails ranges from 0.86 to 1.16, but the initial ratio changes during operation as a result of residual stress relaxation and damage accumulation). This factor should be taken into account in an experimental design.

The selection of a specific lubricating composition from many options should be made during the laboratory testing phase. Therefore, the purpose of this work was to develop a methodology for the express assessment of the effectiveness of a lubricating composition based on a comprehensive analysis of data on changes in the main characteristics of the tribology (wear value and friction coefficient) and strength (hardness value and damage parameter) during an experiment. Therefore, the main focus of this work was on the mechanical aspects of tribosystem behavior.

## 2. Materials and Methods

### 2.1. Experiments on Friction and Wear

**Figure 1:**
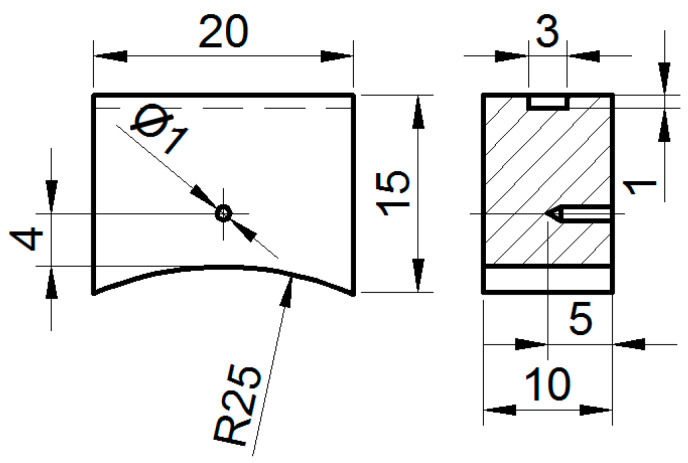
Drawing of a sample with a thermocouple hole for testing (all dimensions are in mm).

Experiments on friction during sliding according to the disk–sample scheme were carried out on a 2070 SMT-1 test rig (RSCVM, Moscow, Russia). The test rig included accessories to automatically record the friction torque and forces, the contact zone temperature, and sample wear, as well as software to automatically process the experimental data. The test samples (Figure 1) were prepared from two types of steel: railroad steel, the samples of which were prepared from the central part of the railhead, and industrial steel, the samples of which were prepared from 20 × 20 mm square rods. A detailed description of the experiments is given in [24].

The counter body (50 mm diameter and 10 mm thick disk) was made from wheel steel. The working parts of the samples (R25, Figure 1) and, after fabrication, the counter bodies were polished in two steps with P1000- and P1500-graded sandpaper according to ISO-6344-2:2021 [25]. The samples were not subjected to special heat treatment.

Flat samples with a 3 × 6 mm section were used to determine the main mechanical properties of the steels (Young’s modulus *E*, yield strength $\sigma_{y.s.}$, and ultimate strength $\sigma_{u}$) obtained from the relevant parts of the rail and wheel rim. Tensile tests were performed on the Bi-00-201 servo-hydraulic test rig (BiSS, Bangalore, India). The mechanical properties of the materials used for the production of nano-powders (M2 copper, MA2 magnesium alloy, and Al-Mn alloy) were also determined. The tensile samples consisted of 3 mm thick plates made from the specified materials. The tensile tests of the samples were carried out in accordance with the National Standard of Ukraine EN 10002-1: 2006 “Metal materials. Tensile tests”. Table 1 shows the chemical composition and main mechanical properties of the steels and materials (except graphite) used for the production of nano-powders. The chemical compositions of all of the materials were determined using the spectrometer ElvaX by ElvaTech Ltd. (Kyiv, Ukraine).

The sample wear was measured using a non-contact ZXE-type displacement sensor (OMRON, Kyoto, Japan) on a small cross-section of each sample. In the process of friction, the samples heated up to 50 ÷ 60 °C. The thermal expansion could be omitted as a result of the small size of the samples. Control measurements of the sample dimensions performed upon the completion of the experiments showed that the errors in the determination of the magnitude of wear on the samples using the non-contact sensor did not exceed 4%.

The following conditions were used in the tests: rotation frequency—300 rpm; normal pressure force—555 N; and continuous operation time—3 h. A computerized registration system recorded the friction torque and force magnitudes, as well as the temperature of the contact zone and sample wear. The coefficient of friction (COF) was determined as follows:

(1) $$f=\frac{2M}{ND}$$

where *M*—friction moment; *D*—counter-body disc diameter; and *N*—normal pressure force.

The initial value of the COF was determined after 5 min from the beginning of the experiments. For different samples, the initial values differed slightly from each other, which was due to the presence of still-undeformed additive particles and a possible difference in the roughness of the working parts of the samples. Therefore, to analyze the effect of various additives on the change in the coefficient of friction, the experimental data are given in relative values $\frac{f}{f_{0}}$, where $f_{0}$ is the initial value of the coefficient of friction and f is its current value.

The wear of the steel samples was investigated under sliding friction conditions with lubrication. AIMOL’s Greaseline Lithium BIO Rail 000 lubricant [26] was selected for this study. This lubricant is currently used for the lubrication of rails on some sections of the South-Western Railway of Ukraine, which is why it was chosen for this research. This biodegradable industrial lubricant is a blend of synthetic polyesters with a lithium thickener. Additives in the form of nano-powders made of the metal materials listed in Table 1, as well as from graphite for the manufacture of GK1-grade slate pencils, were used to make lubricating compositions.

The nano-powders of the metal materials were produced through the electro-erosive dispersion (EED) of material granules in a 40% alcohol medium. The granules of the materials under investigation were made from the machining waste (chips) resulting from sample fabrication. The graphite powder was not further reduced to a smaller particle size.

The EED process consisted of passing a high-intensity pulsed current through a layer of metal granules, which led to their destruction, melting, and even vaporization. As this process took place in a liquid medium, two types of metal microparticles and their oxides were produced, differing in size, morphology, and chemical composition during rapid cooling. Particles with sizes ranging from a few dozen to hundreds of nanometers and with a near-spherical shape crystallized from the melt. The vaporization caused by the rapid cooling of metal resulted in the formation of much smaller particles, often with edge facets, which is characteristic of crystalline formations. The ratio of nano- and ultradispersed particles in the mixture depended on the current intensity, the frequency and duration of pulses (an increase in these parameters contributed to an increase in the amount of nanoparticles), and the type of liquid [13,27]. The general scheme of the unit for the implementation of the specified method is provided in [13]. The production capacity of the unit allowed for the production of nano- and ultradispersed powders of different metals and alloys in sufficient quantities for practical applications. Powders from all of the metal materials except the Al-Mn alloy were obtained under the same operating modes of the unit (the same preset values of current intensity, frequency, and number of pulses). The suspensions obtained using the above method were kept in a fume hood until complete evaporation of the liquid. To obtain a nano-powder of the Al-Mn alloy with a dispersity of 1 to 4 μm, a significantly higher number of pulses was used. The dispersity of the dried powders of the other metal materials ranged from 100 to 300 nm. The dispersity of the graphite powder ranged from 6 to 40 μm.

Lubricating compositions were formulated based on the industrial grease Greaseline Lithium BIO Rail 000 with the addition of nano-powders of rail steel (lubricating composition No. 1), graphite (No. 2), copper M2 (No. 3), the magnesium alloy MA2 (No. 4), industrial steel (No. 5), and the aluminum alloy of the Al-Mn system (No. 6). The content of the additives in lubricating compositions No. 1 to No. 5 was approximately 10% by weight. In composition No. 6, the additive content was ~1% by weight. The authors used one experiment for each lubricant composition to test the proposed express performance assessment method for the compositions containing nano-additives. The lubrication of the contact pair was performed once by applying two drops (approximately 0.15 mL) of the pure lubricant or the lubricating composition on the contact surface of the sample.

As one of the objectives of this study was to determine the possibility of using this technology to create lubricant compositions for the higher wear resistance of heavily loaded friction pairs, additional analyses of the morphological features of the constituent nano-powders were not carried out. Metal particles of these sizes are known [8,9,10,11,12] to have a high plasticity and, when subjected to high contact stresses, to be strongly deformed and reduced to smaller sizes. Therefore, the size and morphology of particles have a certain influence only on the initial value of the friction coefficient, which was demonstrated by the results of the experiments. All of the friction regimes are listed in Table 2 (the + sign means that the experiments were completed).

### 2.2. Methodology for the Assessment of Damage from Friction

During friction processes involving rough surfaces, the hardness of the surface layers of the parts changes due to local plastic deformation. This phenomenon is directly correlated with the wear intensity. However, stable correlations of these processes have not been found, as the experimental data are still scarce and not systematized [28]. Contact interactions of hard and rough bodies are characterized by the discrete and stochastic distribution of surface forces and the sources of heat generation, as well as by high gradients of stress, deformation, and temperature. Accordingly, the surface layers of the material develop a high concentration of defects in the crystalline structure. They are also characterized by specific phase transformations, often involving changes in the chemical composition. The presence of lubrication, additives, and grease materials in the contact zone significantly affects the process. In the case of sliding friction, mainly the surface layers of the material are damaged, and it would be logical to assume that the level of damage can be determined by the degree of hardness dissipation.

The hardness method is the most common method for assessing the condition of a material, and this method features a fairly large number of variants [29]. Due to its physical nature, the hardness should be associated with the material’s mechanical property characteristics during the elastoplastic deformation and destruction. However, most mechanical characteristics are specific to a sample of a particular shape, and the processes of reorganizing the microstructure on the surface and inside the sample differ, even at the stage of uniform deformation, let alone its localization zones. The hardness in relation to the sample dimensions is a local characteristic, not an integral characteristic of materials. Therefore, the known correlations between the hardness and some standard mechanical characteristics can be considered empirical [30,31]. The results of hardness measurements depend on the material, the shape and size of the indenter, the method of application, the magnitude and velocity of the load, the capabilities of hardware to measure the geometric parameters of impressions, the accuracy of the calculation formulas, and so on. Specifically, as the indenter load is reduced, the hardness values increase, and this increase depends on the indenter shape. Ball, pyramid, or cone indentations on a prepared surface of the part are the simplest methods of measuring hardness. However, these methods are characterized by a rather low sensitivity to structural changes in the material caused by the accumulation of microdefects [32], as large volumes of material become deformed compared to the microstructural parameters during the indentation process.

It is obvious that the dispersion of the mechanical properties of many materials is related to the peculiarities of their crystal structure. The hardness could be considered a random value in large-volume tests. Correlations between certain characteristics of the structural state of the material and the parameters of the statistical distribution law of the hardness measurements can be established by reducing the errors associated with the hardness measurement equipment and the so-called human factor, and through using modern automated instruments for research. According to long years of scientific research on the distribution laws of mechanical property characteristics, the results of which are presented in certain review articles [33,34], the hardness dispersion obeys a log-normal distribution, or the Weibull distribution law in the case of small statistical samples. The Weibull law is the preferred option in [34], as this distribution provides only positive values for the random variable, and this corresponds to the ideas in physics related to the characteristics of mechanical properties.

The results of experimental studies on damage accumulation processes in metal materials of different grades under cyclic, short-term, and long-term static loading are presented in [35,36,37,38,39,40]. The damage level was evaluated based on the statistical distribution parameters of the hardness measurement data and the mean hardness value. The correlation coefficient and the homogeneity parameter (the shape parameter of the Weibull distribution) were used as statistical parameters. The fact that the degree of dispersion of the material hardness changes due to any energy effects leading to structural changes in the material can be considered the main conclusion from the analysis of the known experimental data. However, if there were no phase transformations or changes in the chemical composition or density of the material’s surface layers during thermo-mechanical loading, the average hardness value would change only slightly. At the same time, the scatter of hardness measurements would increase with the growth of the operating time parameter, the choice of which would depend on the type of experiment (in uniaxial static tension—stress or strain values, in cyclic loading—number of cycles and maximum cycle stress, and in long-term loading—stress and strain values and creep values) [35,36,37,38,39,40]. The data allowed the authors to substantiate the main idea of the method for an assessment of material damage, which is based on determining the correlation between the statistical dispersion parameters of the hardness measurement during large-volume tests and the structural material’s operating time.

For sliding friction, the value of the sliding path is L=πDn, which, in this case, was equal to ~2830 m/h (the maximum sliding path implemented in the experiments was ~8480 m), and was used to define the operating time. In the experiments in this study, the operating time of the unit may be considered equivalent to the operating time parameter, as the counter-body speed remained unchanged.

In our experiments, the hardness of the sample’s operating part was measured before starting and after completing the experiment. The COMPUTEST SC portable hardness tester manufactured by ERNST (Emdoor Group, Shenzhen, China) was used to measure the hardness (Rockwell, HRC scale). A load of 49 N was applied to the conical diamond indenter with an angle of 110° at the top. The samples were fixed in the desired position with a special device in order to measure the hardness of the sample’s working part. The hardness and damage level of the material at baseline were measured in two areas of ~25 mm^2^ each. The measurement areas were arranged symmetrically in relation to the center section of the sample, with 15 measurements taken in each area. At the end of the experiments, the hardness and damage level were determined within an area of ~50 mm^2^ located in the central section of the sample, where the highest contact stresses were implemented. Therefore, the indentation did not affect the level of damage to the working surface of the samples due to friction.

The level of damage to the surface of the material was evaluated by means of the hardness dispersion value according to the standard in [41]. For this purpose, the sample hardness was measured at 30 points within an area of about 50 mm^2^. The obtained data were then checked for gross errors of measurement using the Smirnov criterion. The average hardness, $\bar{H}=\frac{1}{n}\sum_{i=1}^{n}H_{i}$, of the working surface of the samples was then determined. To estimate the damage value, the hardness measurement data were presented in the form of a series, $lgH_{1},\;lgH_{2},\;\ldots ,\;{lgH}_{n}.$ The mean values of the series members,$\;lg\bar{H\;}=\frac{1}{n}\sum_{i=1}^{n}{lgH}_{i}$, and the mean squared deviation, $S=\sqrt{\frac{1}{n-1}{\sum_{i=1}^{n}\left({lgH}_{i}-lg\bar{H}\right)}^{2}}$, were determined. The level of material damage was estimated with the Weibull homogeneity parameter *m* using the Gumbel formula [42]:

(2) $$m=0.4343\;d\left(n\right)\;{\left[\frac{1}{n-1}\sum_{i=1}^{n}{\left(lgX_{i}-lg\bar{X}\right)}^{2}\right]}^{-\frac{1}{2}}$$

The mean value of a random variable can be determined over a different number of observations *n* (i.e., any sample); hence, relation (2) contains the function *d*(*n*), which is referred to as the standard deviation [42]. The values of this function were calculated by the Computational Laboratory (Columbia University) and are presented in some statistical guides. In our case, d=1.1124 for 30 measurements.

An increase in the data variance and a corresponding decrease in the *m* parameter indicate an increase in the material heterogeneity (in some research work, this parameter is named the homogeneity parameter). A high value of the homogeneity coefficient corresponds to a low level of hardness dispersion, and consequently, a better microstructural organization of the material’s surface layers.

**Figure 2:**
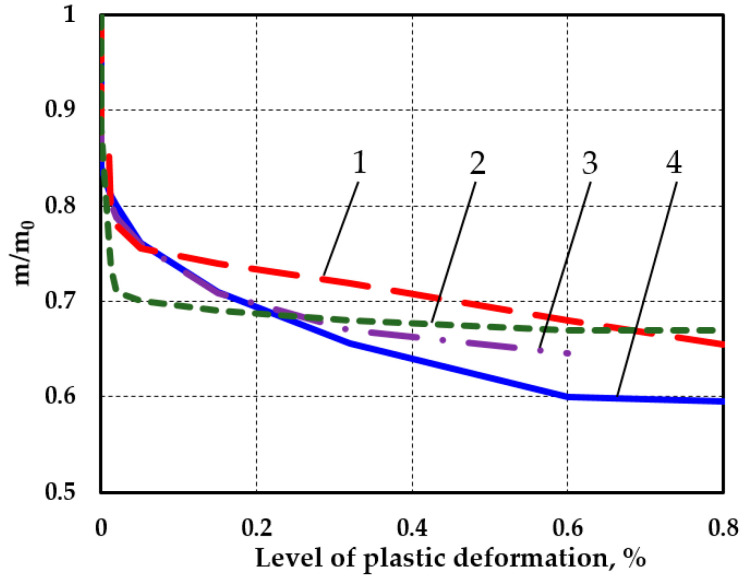
Relationship between degree of hardness dispersion and degree of plastic deformation (1—stainless steel 12Cr18Ni10Ti; 2—industrial steel 0.2%C; 3—Al-Si alloy; and 4—industrial steel 0.45%C).

Note that an increase in the level of hardness heterogeneity, which is considered to represent a certain level of damage to the structure of the material’s surface layers, is not necessarily an indication of damage (i.e., the deterioration of specific operating characteristics). For example, in tensile tests of different materials, the largest changes in the homogeneity parameter occurred at a low elastic–plastic strain, with little effect on changes in the Young’s modulus *E*, the yield strength $\sigma_{y.s.}$, or the strength $\sigma_{u}$. At the same time, in the zone of propagated plastic deformations, the hardness dispersion decreased, but the influence on the above mechanical characteristics increased. Figure 2 shows the relationships between the relative value of the homogeneity coefficient and the magnitude of the deformation of the materials with different grades. The graphs are based on the data provided in [39].

This graph is specifically presented in the article to explain that the degree of plastic deformation of the materials was related to the amount of hardness dispersion. Therefore, it was not by chance that the authors chose this particular parameter (the Weibull homogeneity coefficient m) to assess the damage to friction surfaces.

The mechanical properties or structural condition measurements of the samples characterized the current damage capability of the material $\Pi \left({Τ}_{i}\right)$, where ${Τ}_{i}$ represents certain parameters that characterize the level of the operational load (in our case, it was the length of the sliding path). All materials have a certain initial damage $\Pi_{0}$; therefore, the same method was used for the determination of its level, while the relative parameter $\frac{\Pi \left({Τ}_{i}\right)}{\Pi_{0}}$ was used for the analysis of damage kinetics. The results of the experiments are presented in relative values: $∆H=\frac{\bar{H}-{\bar{H}}_{0}}{{\bar{H}}_{0}}\times 100\%$ and $∆m=\frac{m_{0}-m}{m_{0}}\times 100\%$, where the index 0 corresponds to the initial (conditionally undamaged) state of the material. In its initial state, the ratio of the hardness of the counter body (HRC = 35.3) to the mean hardness of the steel was 1.1 for the rail steel samples and 1.14 for the industrial steel samples.

## 3. Method for Performance Assessment of Lubricating Compositions Containing Nano-Additives

### 3.1. Experimental Research Results

**Figure 3:**
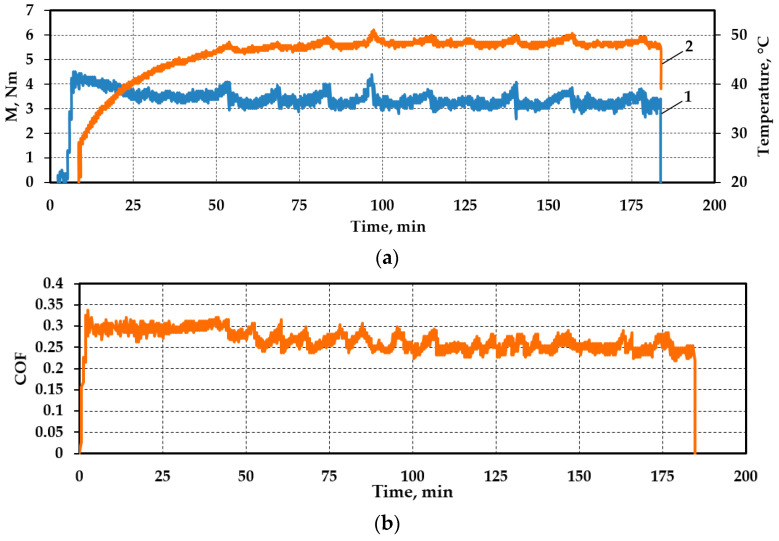
Variation in friction torque (1), temperature (2) (**a**), and coefficient of friction (**b**) for test regime “rail steel with lubricant No. 1”.

The results were obtained in the form of graphs and corresponding numerical arrays; for example, Figure 3 shows the graphs of the variations in the friction torque, temperature, and friction coefficient variations for the test regime “rail steel with lubricant No. 1” (oil with nano-powder of rail steel). In general, some fluctuations for the given test regime caused monotonic changes in the tribological parameters, gradual decreases in the friction torque, and a decrease in the friction coefficient. After the experiment, the final dimensions of the samples were determined using an instrumental microscope. The main results of the experiments are summarized in Table 3.

For the purposes of analyzing the influence of different additives on the change in the COF, the experimental data presented in Table 3 depict the relative values of $f/f_{0}$, where $f_{0}$ represents the initial value of the COF and f represents its value after 1 (upper value) and 3 (lower value) hours of operation.

The results of the experiments (see Table 3) indicate considerable changes in the structural condition of the surface layers of the friction pair material, manifested by changes in the hardness and damage level. It is generally accepted that the amount of wear is proportional to the coefficient of friction. The experiments established that, when using a nano-additive made of steel, which has a lower hardness in the friction pair, the wear is minimal despite a slight increase in the friction coefficient. This suggests that the processes of friction and wear can be controlled to a certain extent through using lubricants of a specific chemical composition and consistency, as well as different additives mixed with them.

**Figure 4:**
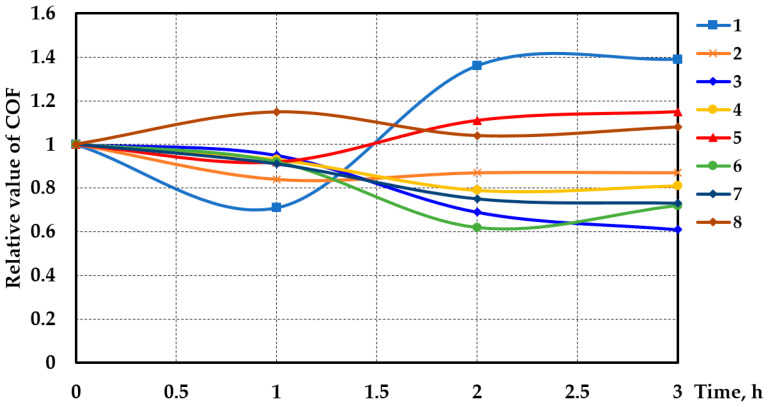
Variation in the relative values of the coefficient of friction over time: 1—rail steel + pure oil; 2—rail steel + lubricant No. 1; 3—industrial steel + pure oil; 4—industrial steel + lubricant No. 2; 5—industrial steel + lubricant No. 3; 6—industrial steel + lubricant No. 4; 7—industrial steel + lubricant No. 5; and 8—industrial steel + lubricant No. 6.

In some of the experiments, a non-monotonic character of COF variation was recorded (Figure 4; curves 1, 5, 6, and 8).

### 3.2. Analysis of the Experimental Results

In [24], a hypothesis was presented about the rationale behind using metal nano-powders, made of the component of the friction pair with a lower hardness, as additives to industrial oils. The hypothesis was experimentally confirmed for the friction pair “rail steel–wheel steel”. In the present work, materials with different chemical compositions were used as additives to an industrial lubricant. The materials also had a lower hardness compared to wheel steel and industrial steel, which were used to make the counter-body disks.

**Figure 5:**
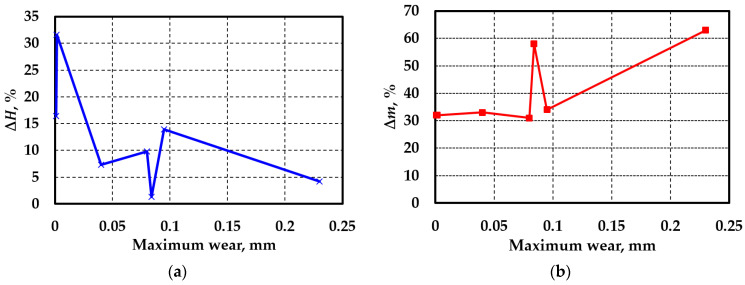
Dependence of the increase in the mean hardness ∆H (**a**) and the homogeneity coefficient ∆m (**b**) on the maximum wear.

The experiments showed that the chemical composition of the additive had a significant effect on the change in the tribological and strength characteristics during friction. For all of the test regimes (see Table 3), there was an increase in the mean hardness and accumulation of damage to the surface layers of the samples, the extent of which depended on the type of additive. To establish the patterns of this influence, the correlations between these characteristics and the value of maximum wear ∆h were explored. According to the results, there was no correlation between the increase in the mean hardness ∆H and the value of maximum wear (Figure 5a). This finding confirmed the conclusions of [35,36,37,38,39,40]. At the same time, the ∆m—∆h dependence can be described by the slightly nonlinear smooth function if the data for the only nonmetallic additive (graphite powder) are disregarded in the series of experiments (Figure 5b). The number of experimental points on the graphs is different, as the data were very similar for compositions No. 1, 4, and 5, and therefore, they do not appear on the adopted scale.

According to Table 3, the greatest increase in the average hardness, the lowest level of damage, and the lowest level of wear were recorded for compositions No. 1, 4, and 5, indicating that these lubricating compositions have certain repair and maintenance properties. This signals that these lubricating compounds possess certain corrective properties.

Considering that these three lubricant compositions are all suitable for wheel and rail lubrication, composition No. 1 (containing the additive of rail steel powder) would be the preferred one as, in addition to a moderate increase in hardness, minimal surface damage, and almost no wear, the optimal value of the coefficient of friction (Figure 4, curve 2) was achieved with this composition, and its value stabilized fairly quickly. Composition No. 5 was based on an industrial lubricant with the addition of industrial steel nano-powder, similar to rail steel in terms of its chemical composition. Therefore, it provided almost the same set of tribological and strength characteristics. Such lubricant additives will not further affect the oxidation of the sample surface, unlike the magnesium alloy additive used to make composition No. 4. Magnesium, as a major component of this additive, will be oxidized due to its higher electrochemical potential compared to iron. Consequently, the properties of such a lubricant composition are likely to be unstable. Furthermore, under these experimental conditions, the additive composition of the magnesium alloy provided too low of a coefficient of friction for wheel and rail lubrication. However, for other friction pairs, it could be effective.

The additive of aluminum alloy in the form of powder with a dispersion of 1 ÷ 4 μm (No. 6) was ineffective under these experimental conditions. Such an additive significantly reduced the friction coefficient. However, this effect was unstable as, in the final stage of the experiment, the COF started to increase, leading to increased wear. The powdered graphite additive (No. 2) did not lead to an increase in the wear resistance, although it led to a reduction in the friction coefficient.

Many researchers consider copper-based additives to be the best option [43,44,45], as such additives create a protective film (called servovite) on the surface of the part, thus contributing to wear reduction. However, in the case of the present study, the maximum wear of the sample was recorded for the composition containing copper powder M2 (No. 3). This suggests that a continuous protective film was not created on the surface of the sample under the respective experimental conditions. This could have been due to the rather large size of the powder particles and their composition, as the EDD processing of copper granules forms particles not only of pure copper, but also of CuO and Cu_2_O oxides, which have different mechanical properties. Similar results were obtained in [46,47], where copper powders of 80–120 nm dispersion were used as an additive to industrial grease. Complex studies in the area of metal physics carried out by the authors of the above-mentioned works have shown that, instead of the servovite film, a thin interface layer containing fragments of the base metal and deformed copper particles as well as iron and copper oxides is created. A sufficiently high value of the homogeneity coefficient (see Table 3) indicated a significant heterogeneity in the hardness distribution, which was obviously associated with the different hardnesses of the interface layer constituents. The mean hardness did not exhibit any considerable variation.

The composition of the industrial oil used as the basis of the lubricating composition also had a strong influence on the wear resistance. The tribological investigation of a synthetic ester base oil with Cu nanoparticles at concentrations of 0.3 and 3.0 wt.% showed that they are not suitable for use as friction modifiers, nor as anti-wear agents, as they significantly increased the wear (up to 7.5 times) [48]. Note that synthetic esters are also the basis of Greaseline Lithium BIO Rail 000 industrial oil.

It is known [46,47,49] that, when powders with a dispersion of 40 μm or greater are used as an additive, a continuous servovite film is not formed. In this case, solid particles contribute to the intensification of the plastic deformation processes of friction surface irregularities, leading to the emergence of fine (subgrain) structures. The powder particles are highly deformed, reduced to smaller sizes, and have a subgrain structure. Some of them fill the microcavities on the friction surfaces, due to which the overall roughness of the surfaces is reduced. This structure improves the strength properties of the material, including the hardness, fracture resistance, and crack resistance, thereby increasing the wear resistance of the friction pairing. These processes undoubtedly depend on the compatibility of the friction pair materials, the lubricating oils, and the nano-powders used as additives. As the chemical composition of additive No. 1 was identical to the friction pair component with a lower hardness, the powder particles joined the base metal (also due to mass transfer processes). This was promoted by a temperature increase in the friction zone.

### 3.3. Lubricant Composition Performance Evaluation Method

The main general requirements applicable to the lubricants used for rails and wheels are a high performance of the contact surface lubrication over a wide temperature range, ease of application, a long residence time in the friction zone, a high contact load resistance, metal corrosion prevention, the lowest possible environmental impact, fire safety, accessibility, and a moderate cost. Industrial lubricating oil-based compositions with nano-powder additives of different chemical compositions are designed taking into account that the additives should not significantly affect the basic properties of the lubricating oil, such as its density, viscosity, adhesion characteristics, and so on. Therefore, the volume content of the additives in the composition should be insignificant.

Criteria have been formulated for conducting performance assessments of lubricating compositions with additives of different chemical compositions for wheel–rail friction pairs under laboratory conditions. The variation in the current relative hardness of the sample surface $∆H=\frac{\bar{H}-{\bar{H}}_{0}}{{\bar{H}}_{0}}\times 100\%$, the variation in the level of current relative material damage $∆m=\frac{m_{0}-m}{m_{0}}\times 100\%$, the current value of wear ∆*h* (in this case, equal to the difference between the initial and current sample sizes in the section of maximum contact stresses), and the current level of the coefficient of friction *f* were chosen as the key parameters for a performance assessment. The variation in these parameters during the friction experiment was analyzed in relation to the generalized operating life (in this particular case, this is the length of the friction path or the duration of the experiment, as they are interrelated, with the friction path being equal to about 8.5 km at an experimental duration of 3 h). Additional parameters for the analysis could include the difference in the electrochemical potentials of key chemical elements of the additive and sample materials, the general cost of the lubricant, and so on.

A high-performance lubricant in relation to the original (for example, a pure lubricant or another composition) should provide less wear, $∆h_{comp}\left(N\right)-{∆h}_{init}\left(N\right)\leq 0$; a lower level of damage, i.e., $∆m_{comp}\left(N\right)-{∆m}_{init}\left(N\right)\leq 0$; and a lower coefficient of friction, i.e., $f_{comp}\left(N\right)-f_{init}\left(N\right)\leq 0$ (the index *comp* denotes the potential composition, while the index *init* denotes the composition chosen for comparison). The latter condition is not mandatory as, in some cases, the value of the coefficient of friction may be subject to certain regulations. The restriction of the choice of additives based on metals, which differ significantly in terms of their electrochemical potential relative to iron, may be introduced as an additional condition. If iron is more active in this kind of galvanic pair (for example, for additives containing copper), then this will accelerate the corrosion of the steel parts in open friction pairs. If the additive material (magnesium, aluminum) is more active, this can lead to a change in its properties after a short period of time. Therefore, additives from materials with an insignificant difference between the electrochemical potential values of the friction pair materials and the additive can be considered feasible.

It should be noted that, for the purpose of correcting such an assessment, it is necessary to compare the results for different lubricating compositions obtained under the same experimental conditions. According to the given criteria, lubricating composition No. 1 (industrial grease containing a rail steel nano-powder additive) was effective for use in heavily loaded friction pairs of the wheel–rail type. This composition had corrective properties, provided an optimal value of the friction coefficient, and resulted in minimal damage and wear to the friction surface.

Moreover, such an additive is nontoxic and has a moderate cost, as it can be produced from metal waste generated during the mechanical processing of rails. The chemical composition of such an additive is close to the chemical composition of wheel steels; therefore, its use will have a positive effect on improving the wear resistance of both rails and wheels. For this reason, it can be recommended for the lubrication of the parts of turnouts, as well as the rails and the wheels of the rolling stock.

For friction experiments where the determining factor is abrasive wear, the proposed express method allows complex physical studies of damage to the surface layers of the material to be replaced with simpler mechanical hardness tests at the stage of a preliminary assessment of the effectiveness of the lubricant. Naturally, the promising lubricant compositions selected in this stage should be further studied in detail using physical methods, as well as under natural conditions on the railway.

## 4. Conclusions

This work considered an express method for the quantitative and qualitative assessment of the effectiveness of lubricating compositions with nano-additives of different chemical compositions for increasing the wear resistance of steel friction pairs. The studied method consisted of a joint analysis of the kinetics of damage accumulation and changes in the mechanical and tribological characteristics during sliding friction. The parameters of the statistical distribution of the hardness measurements in serial tests were used to assess the level of damage to the material. In particular, this statistical parameter could be the shape parameter (homogeneity parameter) of the Weibull distribution.

The variation in the current relative hardness of the sample surface, the variation in the level of current relative material damage, the current value of wear, and the current value of the coefficient of friction were chosen as the key parameters for the performance assessment.

The influence of other experimental parameters that affected the sliding friction processes, such as the value of the load, the level of contact stresses, the temperature, the surface roughness, and so on, can also be assessed according to the criteria mentioned. If certain variation limits of the generalized operating time indicator are indicated, then the feasibility of continuing the experiments may be considered depending on the fulfillment of these conditions at the end of each section. This kind of assessment system can obviously be supplemented with other criteria.

The results of the experiments on sliding friction with the lubrication of steel friction pairs were used to support the method. The lubricating compositions were based on Greaseline Lithium BIO Rail 000 industrial oil and additives comprising copper nano-powders, aluminum and magnesium alloys, graphite, and two grades of medium-carbon steels.

According to the indicator framework presented in this paper, the most effective composition for increasing the wear resistance of the steel friction pairings was the industrial lubricant with the addition of rail steel nano-powder. This composition offered the optimal coefficient of friction, minimal damage and wear to the friction surface, and a moderate cost.

The suggested express method enables the identification of the corrective properties of lubricating compositions with nano-additives in laboratory studies. Therefore, the methodology presented for integrated performance analyses is expected to be useful for rapidly assessing the influence of lubricants on the wear resistance of friction pairs.

## Figures and Tables

**Table 1:** Chemical composition and main mechanical properties of test materials.

Materials	Chemical Composition (In Terms of Main Components)	E, GPa	$\sigma_{y.s.}$, MPa	$\sigma_{u}$, MPa
Rail steel	C—0.57%, Si—0.32%, Mn—0.94%, Fe—base	211	740	920
Industrial steel	C—0.18%, Si—0.19%, Mn—0.41%, Cr—0.05%, Fe—base	210	250	435
Wheel steel	C—0.58%, Si—0.34%, Mn—0.76%, Fe—base	212	845	985
M2 copper	Fe—0.04%, Ni—0.08%, Sn—0.02, Cu—base	117	116	210
MA2 magnesium alloy	Al—4.32%, Mn—0.32%, Zn—1.16%, Mg—base	48.8	135	230
Al-Mn alloy	Mn—1.24%, Si—0.26%, Fe—0.18%, Cu—0.09, Al—base	70.5	141	224

**Table 2:** Friction regimes investigated.

Sample Material	Lubricant Composition		
	Pure Oil	No. 1	No. 2–No. 6
Rail steel	+	+	−
Industrial steel	+	−	+

**Table 3:** Key results of the experiment.

Sample Material	Friction Mode, Lubricating Composition Number	Maximum Wear	Initial Coefficient of Friction	Relative Coefficient of Friction	Variation in Mean Hardness	Variation in Homogeneity Coefficient
		∆h, mm	$f_{0}$	$f/f_{0}$	∆H, %	∆m, %
Rail steel	Pure oil	0.08	0.22	0.71/1.39	+9.8	+31
	No. 1	<0.001	0.29	0.84/0.87	+14.7	+29
Industrial steel	Pure oil	0.095	0.35	0.95/0.61	+13.9	+34
	No. 2	0.084	0.26	0.93/0.81	+1.3	+58
	No. 3	0.23	0.22	0.92/1.15	+4.2	+63
	No. 4	0.0015	0.22	0.92/0.72	+31.6	+32
	No. 5	0.001	0.35	0.91/0.73	+16.4	+32
	No. 6	0.04	0.25	1.15/1.08	+7.3	+33

## Data Availability

The original contributions presented in the study are included in the article, further inquiries can be directed to the corresponding authors.
